# Loss of interleukin 33 expression in colonic crypts - a potential marker for disease remission in ulcerative colitis

**DOI:** 10.1038/srep35403

**Published:** 2016-10-17

**Authors:** Mona Dixon Gundersen, Rasmus Goll, Johanna Hol, Trine Olsen, Renathe Rismo, Sveinung W. Sørbye, Olav Sundnes, Guttorm Haraldsen, Jon Florholmen

**Affiliations:** 1 Research Group of Gastroenterology and Nutrition, Department of Clinical Medicine, UiT The Arctic University of Norway, and the University Hospital of North Norway, Tromsø, Norway; 2 Dept. of Pathology and Jebsen Inflammation Research Centre, University of Oslo and Oslo University Hospital Rikshospitalet, Oslo, Norway

## Abstract

Interleukin 33 (IL-33) is a cytokine preferentially elevated in acute ulcerative colitis (UC), inferring a role in its pathogenesis. The role of IL-33 in intestinal inflammation is incompletely understood, with both pro-inflammatory and regulatory properties described. There are also conflicting reports on cellular sources and subcellular location of IL-33 in the colonic mucosa, justifying a closer look at IL-33 expression in well-defined clinical stages of UC. A total of 50 study participants (29 UC patients and 21 healthy controls) were included from a prospective cohort of inflammatory bowel disease patients treated to disease remission with infliximab, a tumour necrosis factor alpha (TNF) inhibitor. To our knowledge this is the first study examining mucosal IL-33 expression before and after anti-TNF therapy. In colonic mucosal biopsies we found a 3-fold increase in IL-33 gene expression comparing acute UC to healthy controls (p < 0.01). A significant reduction of IL33 between acute UC and disease remission was observed when TNF normalised in the mucosa (p = 0.02). Immunostaining revealed IL-33 in the nuclei of epithelial cells of scattered colonic crypts in acute disease, while at disease remission, IL-33 was undetectable, a novel finding suggesting that enterocyte-derived IL-33 is induced and maintained by inflammatory mediators.

Ulcerative colitis (UC) is a chronic inflammatory bowel disease (IBD) characterised by a continuous, submucosal inflammation of the colon. The pathogenesis is complex and appears to involve both genetic susceptibility and environmental triggers, that together lead to an imbalance between pro- and anti-inflammatory cytokines in the intestinal mucosa and development of chronic inflammation^1^,^2^. This assumption is supported by the success of inhibiting the pro-inflammatory cytokine tumour necrosis factor alpha (TNF), which has proven effective in achieving mucosal healing in IBD^3^,^4^.

Interleukin 33 (IL-33), a member of the interleukin-1 family of cytokines, has generated interest in the research field of IBD following reports of its dysregulation^5^,^6^,^7^,^8^,^9^. Notably, IL-33 is predominantly elevated in acute IBD and most markedly in UC^6^,^7^,^8^,^10^,^11^,^12^. In addition, genetic polymorphisms of IL-33 or its receptor, IL1RL1 (alias IL-33R, ST2), are associated with an increased risk of IBD and a more extensive colitis^9^. In experimental colitis, IL-33 is found to induce both pro-inflammatory and tissue protective pathways^6^,^13^,^14^,^15^,^16^,^17^,^18^, the latter considering a crucial role for IL-33 in promoting tissue healing and raising the interest of IL-33 as a potential therapeutic target in IBD^16^,^17^.

IL-33 was initially discovered as a nuclear factor of high endothelial venules^19^. A wide range of cells have since been found to express IL-33, including cells at sites protecting the body from the outer environment such as the skin, airways and gastrointestinal tract^13^,^20^. The term “alarmin” is used to describe the role of IL-33 when released from injured or necrotic cells in response to tissue damage^21^,^22^. When released, IL-33 acts as a potent activator of the intestinal immune system by binding the transmembrane isoform of the IL-33 receptor IL1RL1. The IL-33/IL1RL1 complex activates NF-κB (nuclear factor kappa-light-chain-enhancer of activated B cells) and MAPK (mitogen-activated protein kinases), including induction of a TH2 cellular response in T cells^5^,^22^,^23^. Recent findings also suggest that IL-33 promotes the differentiation of T regulatory cells in the intestine associated with protection against dysregulated inflammatory responses^16^. Murine models with DSS (dextran sulphate sodium) -induced colitis support the hypothesis of IL-33 as a dual function cytokine. Exogenous treatment with IL-33 has been shown by several independent groups to increase the severity of acute colitis^6^,^13^,^15^,^24^. Interestingly, mice deficient of IL-33 have a delayed recovery and resolution after an induced colitis episode^18^. In contrast to IL-33 therapy exacerbating DSS-colitis, a recent report found IL-33 treatment to promote epithelial repair driven by innate lymphoid type 2 cells and regenerative growth factors^17^. Taken together, this implies that the role of IL-33 in the pathogenesis of ulcerative colitis varies according to phases of inflammation and mucosal healing.

The aim of the current study was to characterize the modulation of IL-33 in the intestinal mucosa of patients with acute UC treated with infliximab, a TNF inhibitor, and to unravel the dynamics of IL-33 in the mucosal healing process. To our knowledge this is the first study that examines the mucosal response of IL-33 following anti-TNF therapy in IBD.

## Results

### Study population

Fifty study participants were included, 29 UC patients and 21 healthy controls. Baseline demographics for the study population are shown in Table 1.

### Mucosal levels of IL33 mRNA are increased in acute UC

**Figure 1:**
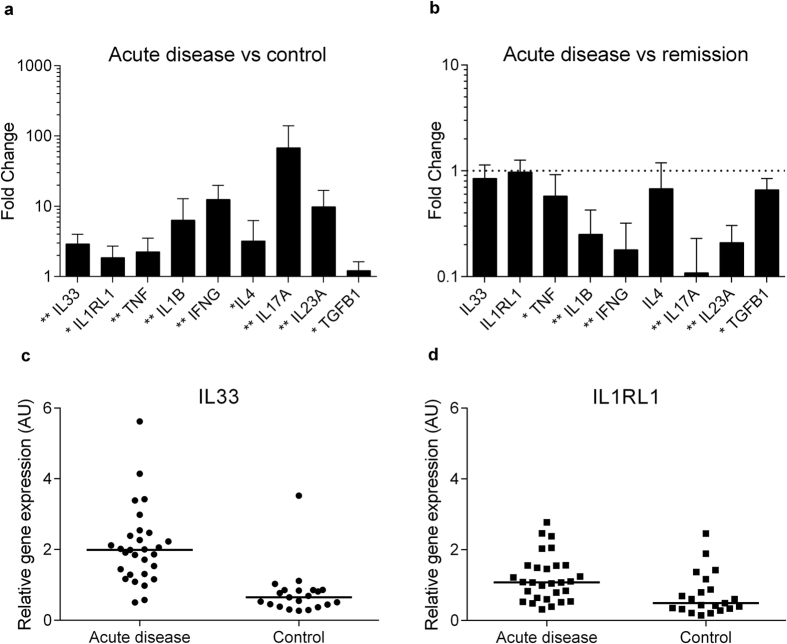
Expression of mucosal IL33 is increased in acute ulcerative colitis. Panel (a) shows an increase in mucosal mRNA expression for selected cytokines from colonic biopsies comparing acute ulcerative colitis (n = 29) with a healthy control group (n = 21). Panel (b) shows the same cytokine profile for patients with ulcerative colitis (n = 29) with a pairwise comparison of biopsies taken during active disease and at disease remission. No change was detected for IL33, IL1RL1 and IL4 (p > 0.05). Results for (**a**,**b**) are given as fold change (2^−∆∆CT^) with mean values, and the upper limit of the 95% confidence interval as error bars. Statistical analysis was performed using Mann U Whitney Test (**a**) and Wilcoxon Signed Ranks Test (**b**) with *p-value < 0.01, **p-value < 0.001 shown in panels. Panels (c) and (d) show individual values of relative gene expression (2^−∆CT^) of IL33 and IL1RL1, respectively, for acute disease and normal controls, with the horizontal line representing the median value.

Quantitative PCR analysis revealed a 3-fold increase of mucosal IL33 transcripts in biopsies from acute UC lesions compared to healthy control samples (p < 0.001, Fig. 1a). Expression of the IL33 receptor IL1RL1 was also upregulated, as were several other pro-inflammatory cytokines shown in Fig. 1a. Data from individual patients are shown for IL33 and IL1RL1 (Fig. 1c,d, respectively). We next assessed the modulation of this cytokine profile in each patient before and after infliximab therapy (acute disease versus remission), observing a strong reduction in several cytokines, whilst changes in transcript levels of IL33 or IL1RL1 did not reach statistical significance (Fig. 1b).

### IL33 mucosal gene expression decreases when TNF levels normalise

**Figure 2:**
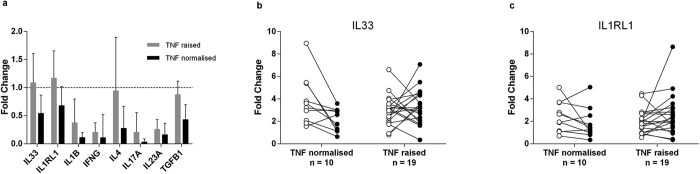
Normalisation of TNF reduces mucosal IL33 gene expression. Pairwise comparison of colonic biopsies from patients with UC pre and post infliximab therapy (acute disease versus disease remission) were analysed with real-time qPCR. Patients are grouped into those who showed normalisation of mucosal TNF at disease remission (n = 10), and those who had a raised mucosal TNF at disease remission (n = 19). Results are given as mean fold change (2^−∆∆CT^) with error bars marking the upper limit 95% confidence interval. A more significant reduction of inflammatory cytokine transcription was seen in the group with normalisation of mucosal TNF, assessed by Wilcoxon Signed Ranks Test (**a**). Panels (b,c) show individual IL33 and IL1RL1 gene expression for each patient at acute disease (white point) and disease remission (black point) given as 2^−∆CT^, with ∆CT values generated from the mean of the healthy control group. IL33 showed a significant reduction (p = 0.02) for the TNF normalised group but not when TNF remained elevated (p = 0.8).

The lack of reduction in IL33 gene expression upon disease remission was somewhat surprising given the association of IL33 expression with a pro-inflammatory cytokine profile. We therefore stratified our cohort according to TNF-normalisation (defined as the upper limit of the 95% confidence interval of a previously published normal control group)^25^,^26^, resulting in two subgroups; those with normalised TNF mucosal levels (n = 10) and those with a raised mucosal TNF (n = 19). Cytokine expression was compared between these subgroups shown in Fig. 2a. A significant reduction of IL33 mRNA was only seen in the normalised TNF group (p = 0.02, see Fig. c), whereas we observed no difference with respect to IL1RL1 (Fig. 2a,c).

### Loss of nuclear IL-33 in epithelial crypt cells in healed mucosa

**Figure 3:**
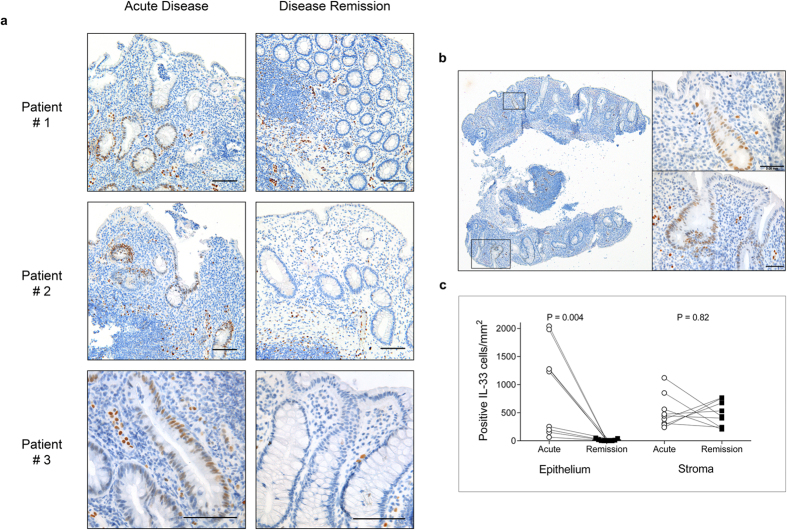
IL-33 is present in epithelial crypts in acute ulcerative colitis. Panel (a) shows colonic biopsies from 3 representative patients with acute UC treated with infliximab (anti-TNF) to disease remission. Endoscopic biopsies were taken from the most inflamed colonic regions pre- infliximab therapy (acute disease), and repeated from the same region at disease remission. Biopsies were formalin fixed, paraffin embedded with immunoenzymatic staining for IL-33 (brown) using monoclonal mouse antibody (Nessy-1). IL-33 positive cells are seen in epithelial crypts during acute disease. At disease remission no epithelial staining for IL-33 was seen. Cell nuclei (blue) counterstained with hematoxylin. Scale bar = 0.1 mm. Panel (b) shows a representative whole biopsy from acute ulcerative colitis (x40 magnification) stained as above, whilst boxes show high-power magnification. Scale bar = 0.05 mm. Panel (c) shows quantification of IL-33 positive cells per mm^2^ in individual patients (n = 9) in the epithelium and lamina propria.

Observing that IL33 transcription was only reduced in patients with normalised TNF levels, we next assessed the expression level of IL-33 protein by means of immunohistochemical analysis. Nine UC patients with mucosal biopsies taken from the same region at both acute inflammation (pre-infliximab) and at disease remission were included. In lesions of acute inflammation, 8 out of 9 biopsies showed a positive, nuclear IL-33 signal in epithelial cells in scattered colonic crypts. We further investigated whether these crypts were associated with crypt abscesses or inflammatory cell clusters, but did not detect any distinct pattern. Strikingly, IL-33 was undetectable in all colonic crypts and epithelial cells at the time point defined as disease remission following infliximab therapy (See Fig. 3). Interobserver agreement of quantification of IL-33 positive cells yielded a weighted kappa coefficient of 0.83 (data shown in Supplementary Table S1).

### IL-33 in the lamina propria

**Figure 4:**
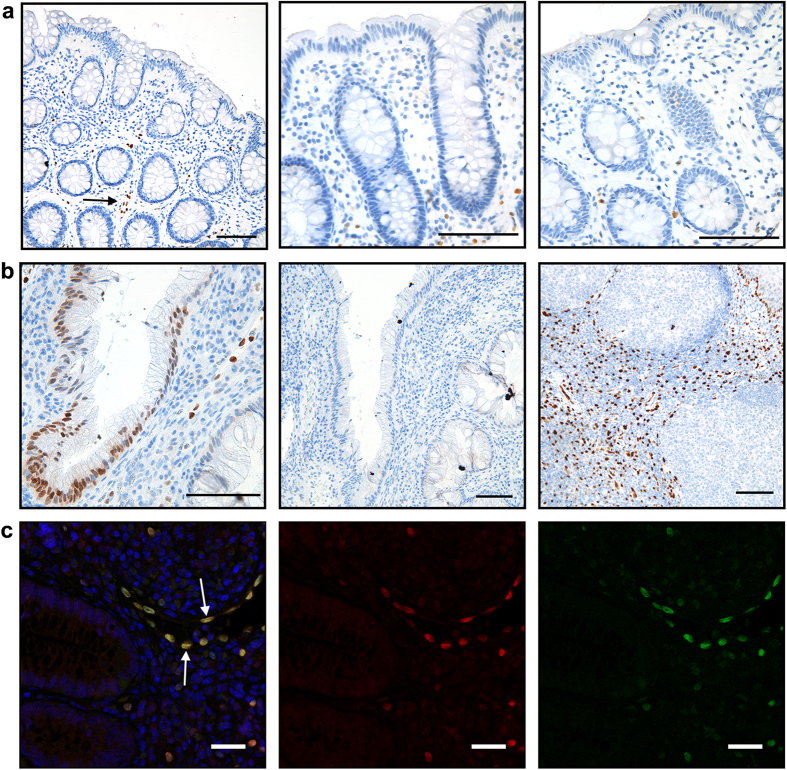
Immunostaining of IL-33 in control groups. Panels (a,b) show formalin fixed, paraffin embedded biopsies with immunoenzymatic detection for IL-33 (brown) using monoclonal mouse antibody (Nessy-1) at [1 μg/ml], with cell nuclei (blue) counterstained with hematoxylin. Panel (a) shows representative sections from colonic biopsies of 3 healthy controls. No IL-33 staining was observed in epithelial cells. IL-33 positive cells are seen in the stroma mainly associated with vascular endothelial cells (black arrow). Scale bar = 0.1 mm. Panel (b) shows from left to right; acute UC with a IL-33 positive colonic crypt, an isotype and concentration matched control from the same biopsy and a positive control using human tonsillar tissue. Scale bar = 0.1 mm. Immunofluorescence images (**c**) of formalin fixed, paraffin embedded colon biopsies from acute UC, with double staining performed for IL-33 polyclonal goat antibody (red) and IL-33 monoclonal mouse antibody (green). Merged image (left) shows good co-localisation of both antibodies (see white arrow), confirming antibody specificity. Cell nuclei are counterstained with Hoechst. Scale bar = 20 um.

IL-33 expression in the lamina propria of UC lesions was mainly found in vascular endothelial cells, and in myofibroblast-like cells^7^,^11^. Quantification of IL-33 positive cells in this compartment showed no change in the number of positive IL-33 cells in the lamina propria comparing acute UC with disease remission (Fig. 3c). In the normal control group (n = 10), only a few IL-33-positive lamina propria cells were observed, most of them morphologically assessed as vascular endothelial cells consistent with previous reports^7^,^27^. No IL-33 expressing cells were observed in the epithelial border or colonic crypts of the normal control group (Fig. 4).

## Discussion

In this study we observed a selective induction of nuclear IL-33 in enterocytes in scattered crypts of acute UC lesions. Strikingly, this expression was lost following successful anti-TNF therapy to disease remission. These are novel findings providing insight into a dynamic expression of IL-33 in well-defined, clinical stages of UC.

Although IL-33 is consistently found to be elevated in acute UC, there is variation in the literature regarding its cellular location^6^,^7^,^8^,^28^,^29^, particularly in the epithelial intestinal barrier^6^,^7^,^8^,^29^. In this study we found that IL-33 was present in nuclei of scattered epithelial crypt cells of patients with acute UC, while it was undetectable in disease remission and in healthy controls. These findings suggest that IL-33 may be an inflammation-induced factor in the human intestinal epithelial barrier. In support of this, recent studies at other epithelial barrier sites have described nuclear IL-33 to be associated with inflammation inferring a common pathway of IL-33 regulation, including squamous epithelium cells of the oesophagus, and keratinocytes of the skin epithelial barrier^30^,^31^. We hypothesised that the sporadic distribution of IL-33 positive intestinal crypts might be associated with areas of more severe inflammation such as crypt abscesses, proximity to epithelial injury or aggregates of inflammatory cells, however we did not observe any distinct pattern. Differences in biopsy orientation must be taken into account and may have underestimated our findings of IL-33 positive cells. Another hypothesis is that enterocyte-derived IL-33 is induced by local environmental factors in the individual lumen of colonic crypts, resulting in the scattered distribution observed.

IL-33 is described as a cytokine of dual functions, referring to its role as an endogenous alarmin, but also to its nuclear, transcriptional repressive functions^21^. We found IL-33 to be present in epithelial cells in association with inflammation, however our data do not imply whether IL-33 may act as an inducer or repressor of the inflammatory response in this setting. Furthermore, the mechanisms regarding its downregulation, degradation or secretion from the nuclei remain unknown. Future research should be designed to understand which inflammatory mediators induce and regulate IL-33 expression in crypt cells, and is an area of focus in our research group.

IL33 mucosal gene expression was increased in acute UC, alongside other known mediators of acute inflammation including TNF. The normalisation of mucosal TNF-expression has previously been associated with long-term disease remission in UC^32^. Interestingly, we found a more pronounced downregulation of inflammatory cytokines in the subgroup of patients (n = 10) whom achieved normalisation of mucosal TNF levels, including a significant reduction of IL-33 (p = 0.02). In contrast, IL33 remained upregulated in the patient group with raised TNF levels at clinical remission (see Fig. 2). These findings may indicate a time-lag for normalisation of IL33 in the intestinal mucosa, possibly dependent on a more complete normalisation of pro-inflammatory cytokines including mucosal TNF. The IL33 receptor IL1RL1 showed a similar tendency, however this must be interpreted with care as our assay did not differentiate between membranous and soluble isoforms of IL1RL1, an issue that should be addressed in future studies. From the above findings we propose that mucosal IL33 expression is only reduced at the stage of remission that allows for normalisation of mucosal TNF levels.

In this study, we have described a cohort of patients with a clinically similar disease course, all of whom were followed from acute UC to disease remission during anti-TNF treatment. Biological variation was reduced by comparing serial biopsies from the same patient. We were also able to examine the mucosal IL-33 response to modulation of TNF *in vivo*. In contrast to the significant reduction in epithelial IL-33 observed in response to anti-TNF treatment, we could not detect any reduction of IL-33 positive lamina propria cells (see Fig. 3). Several factors may be contributing to this result, including different cellular regulation of IL-33 expression. Endothelial cells, observed to be a main source of IL-33 positive cells in disease remission, show constitutive Notch-dependent IL-33 expression and this regulatory mechanism has not been described in other cell types^33^. Other factors include sample size (n = 9) and that endoscopic biopsies only sample the superficial layer of the colonic mucosa, excluding deeper stromal layers from our analyses.

Achieving disease remission is associated with a better prognosis in UC, and is the main goal of therapy^34^. Unresolved questions include when therapy can be successfully discontinued following achievement of disease remission. Based on previous findings we propose that clinical and endoscopic criteria are not sufficient for determining disease remission, and that evidence of mucosal normalisation of inflammatory mediators is also needed^26^,^32^.

## Conclusion

We have characterized the expression of IL-33 in well-defined clinical stages of ulcerative colitis. IL-33 was present in the nuclei of enterocytes in scattered colonic crypts in acute ulcerative colitis, but was not present in these cells at clinical disease remission. An overall reduction in IL-33 mucosal gene expression was only seen in patients with normalised mucosal TNF levels. Further studies to explore the regulation and nuclear role of IL-33 are warranted, taking cellular location into account. The downregulation of epithelial IL-33 expression may potentially serve as a marker for disease remission in UC together with other biomarkers including mucosal TNF.

## Materials and Methods

The study protocol, including storage of biological samples, was approved by the Regional Committee for Medical and Health Research Ethics North (Ref no: 1349/2012), and carried out in accordance with the Declaration of Helsinki Principles. Written and informed consent was obtained from all study participants.

### Study population and inclusion criteria

Patients were recruited from a prospective study of IBD patients at the University Hospital of North Norway, aged ≥ 18 years. Mucosal biopsies were obtained from patients with moderate to severe UC defined by a Mayo score ≥ 6 at baseline^35^. Patients were diagnosed according to established clinical, endoscopic and histological criteria^36^. All patients were treated with infliximab (anti-TNF therapy) intravenously 5 mg/kg at 0, 2 and 6 weeks, and every 4th week until disease remission, defined as a Mayo score ≤ 2 with a endoscopic subscore of 0 or 1. Exclusion criteria included anti-TNF therapy within the last 12 weeks, pregnancy, previous cancer or primary sclerosing cholangitis. The control group consisted of patients referred to endoscopy at the outpatient clinic for bowel cancer screening. For this group, exclusion criteria were diarrhoea and presence of colonic polyps. All controls had a normal colonoscopy and a histological normal colonic mucosa assessed by two pathologists.

### Colonic biopsies

Endoscopic mucosal biopsies were obtained from the most inflamed bowel region prior to start of infliximab therapy, and a new biopsy obtained from the same region at disease remission. Mucosal biopsies were taken from the rectum or sigmoid colon in healthy controls. A standard endoscopic biopsy forceps was used for mucosal biopsies which were immediately immersed in either 10% formalin or RNA*later* (Ambion Inc., Austin, TX, USA). RNA was isolated using either the Promega method (Promega Corp., Madison, WI, USA) previously published in detail^26^,^37^, or with AllPrep RNA/DNA miniKit using Qiacube (Qiagen, Hilden, Germany), and stored at −70 °C.

### Quantitative PCR analysis

Reverse transcriptase was performed with Quantitect 2 step Kit (Qiagen, Hilden, Germany) according to the manufacturer’s instructions. Mucosal cytokines were measured in duplicates using hydrolysis probes. Beta actin (ACTB) was used as a reference gene for normalisation. The stability of ACTB in the current setting has previously been tested^26^. Positive and negative controls were included on each plate with standardised cycle thresholds. Assays, apart from interferon gamma (IFNG) were all in-house and listed in Supplementary Table S2. All data was expressed using ∆CT values with fold change expressed as 2^−∆∆CT^. The laboratory investigators were blinded to the clinical data.

### Immunostaining

Sections (4 μm) of formalin-fixed, paraffin-embedded biopsies were deparaffinised and rehydrated. Antigen retrieval was performed in citrate buffer pH 6 in a water bath at 100 °C for 20 minutes, followed by 20 minutes cooling. IL-33 monoclonal mouse antibody (Nessy-1, Enzo life Sciences) was used at 1 μg/ml, or a polyclonal goat antibody for IL-33 at 0.1 μg/ml (AF3625, R&D Systems). Primary antibodies were incubated for 60 minutes at room temperature or overnight at 4 °C. Enzymatic detection with 3,3 diaminobenzidine was performed with DAKO envision kit (DAKO, Glostrup, Denmark) following the manufacturer’s instructions and counterstained with hematoxylin. Negative controls were performed routinely with the primary antibody being substituted with an isotype- and concentration-matched antibody (mouse IgG1, DAKO). Tonsillar tissue was used as positive control. For immunofluorescence detection slides were blocked with 10% donkey serum (20 minutes). Secondary antibodies conjugated with Alexa488 or Alexa546 (Life Technologies) were used, with Hoechst (33258, Life Technologies) as counterstain.

Images where obtained using Olympus BX51 microscope with an Olympus U-TVO.5XC camera using AnalySIS 3.2 software (Soft Imaging System). Immunofluorescence images were taken with a Zeiss Axio observer, Z1 LSM780 CLSM system (Carl Zeiss Microscopy GmbH, Jena, Germany) running the Zen 2012 (black edition) software. Images were processed in Adobe Photoshop CS6 (Adobe Systems Software Ireland Ltd, Dublin, Ireland), with adjustments to image histograms applied only for whole images. Two independent observers (SWS, MDG) performed quantification of IL-33 positive cells. 3 representative high field views of x 400 magnification per biopsy where counted for positive and negative epithelial and crypt cells and summarised in the following categories: negative <1%, weak 1–9%, moderate 10–24%, high 25–49% and very high ≥50%. Positive cells in the stroma were counted per mm^2^ based on biopsy area calculations from NIS- elements AR 3.1 software (Nikon Instruments, Europe).

### Statistical methods

All statistics are performed using IBM SPSS statistics version 22.0 (IBM corp., Armonk, NY, USA) or GraphPad Prism version 6.03 (La Jolla, CA, USA). Normality plots were assessed with histograms and Shapiro-Wilks test, with appropriate t-test or equivalent non parametric test used. Interobserver variability was assessed with weighted Cohen’s kappa for immunohistochemistry analysis.

## Additional Information

**How to cite this article**: Gundersen, M. D. *et al*. Loss of interleukin 33 expression in colonic crypts - a potential marker for disease remission in ulcerative colitis. *Sci. Rep.* **6**, 35403; doi: 10.1038/srep35403 (2016).

## Supplementary Material

Supplementary Information

## Figures and Tables

**Table 1:** Study population baseline demographics.

Baseline characteristics	Ulcerative colitis n = 29	Control group n = 21
Female/male	14/15	8/13
Age study inclusion (years), mean ± SD	39.0 ± 12.7	56.1 ± 13.4
Disease duration, median months, [range]	58 [0–372]	
Newly diagnosed <1month	2	
Disease extent (n)		
- *Proctitis*	5	
* - Left colitis*	17	
* - Pancolitis*	7	
Smoking status (n)		
* - Never*	15	
* - Current*	2	
* - Ex smoker*	11	
* - Unknown*	1	
UCDAI pre, median,[range]	10 [7–12]	
Endoscopic subscore, median, [range]	2 [2–3]	
Medication (last 3 months) (n)		
* - 5ASA*	28	
* - Steroids (oral)*	20	
* - Azathioprine or Methotrexate*	11	
