# Supplementary material for: Loss of interleukin 33 expression in colonic crypts - a potential marker for disease remission in ulcerative colitis
Source: Sci Rep. 2016 Oct 17;6:35403. doi: 10.1038/srep35403 (PMC5066310; doi:10.1038/srep35403)
Supplement: Supplementary Information [file srep35403-s1.pdf]

Supplementary information

Loss of interleukin 33 expression in colonic crypts - a potential marker for disease remission in ulcerative colitis.

Mona Dixon Gundersen[1], Rasmus Goll[1], Johanna Hol[2], Trine Olsen[1], Renate Rismo[1], Sveinung W. Sørbye[1], Olav Sundnes[2], Guttorm Haraldsen[2] & Jon Florholmen[1].

Table S1. Interrater variability.

a

	**Rater 1**					
**Rater 2**	**0 (<1%)**	**1 (<10%)**	**2 (<25%)**	**3 (<50%)**	**4 (>50%)**	**Total**
**0 (<1%)**	58	2	0	0	0	60
**1 (<10%)**	3	7	0	0	0	10
**2 (<25%)**	0	2	0	0	0	2
**3 (<50%)**	0	0	2	3	2	7
**4 (>50%)**	0	0	0	3	2	5
**Total**	61	11	2	6	4	84

b

	**0 (<1%)**	**1 (<10%)**	**2 (<25%)**	**3 (<50%)**	**4 (>50%)**
**0 (<1%)**	1	0.75	0.5	0.25	0
**1 (<10%)**	0.75	1	0.75	0.5	0.25
**2 (<25%)**	0.5	0.75	1	0.75	0.5
**3 (<50%)**	0.25	0.5	0.75	1	0.75
**4 (>50%)**	0	0.25	0.5	0.75	1

Table S1. Interrater variability
Interrater variability was determined with a weighted Cohen's kappa yielding a coeefficient of 0.83 indicating good agreement between raters. Table **a** shows the rawdata for quantification of n= 84 representative slides divided into 5 groups according to percentage of positive interleukin 33 cells. Table **b** shows the weights given for categories.

Supplementary Table S2. Primer sequences.

Genebank ID (HGNC)	Forward primer sequence 5'-3'<br>Reverse primer 5'-3'	Probe sequence 5'-3'	Reference sequence
**IL33**	TGAGTCTCAACACCCCTCAAATG<br>GGCATGCAACCAGAAGTCTTTT	CAGGTGACGGTGTTGATGGTAAGATGTTAATG	NM_033439.3
**IL1RL1**	ACACCTGTAAATTTATACACAATGA<br>CCTTGCTCATCCTTGACC	CCTGGTCGCCGTCACACTAT	NM_NM_016232.4
**TNF**	CACGCTCTTCTGCCTGCTG<br>GATCTGACTGCCTGGGC	CCAGAGGGAAGAGTTCCCCAGGGAC	NM_000594.3
**ILIB**	CCTGAGCTCGCCAGTGAAA<br>TTTAGGGCCATCAGCTTCAAA	ATGGCTTATTACAGTGGCAATGAGGATGACT	NM_000576.2
**IL4**	CGGCTCGACAGGAACCTCT<br>TCCAAGAAGTTTTCCAACGTACTCT	CGGGCTTGAATTCCTGTCCTGTGAAG	NM_000589.3
**IL17A**	TGATTGGAAGAAACAACGATGACT<br>ATTGTGATTCCTGCCTTCACTATG	TGGTGTCACTGCTACTGCTGCTGAGC	NM_002190.2
**IL23A**	CCCAAGGACTCAGGGACAAC<br>TCCTAGCAGCTTCTCATAAAAAATCA	TCAGTTCTGCTTGCAAAGGATCCACCAG	NM_016584.2
**TGFB1**	CTGCTGAGGCTCAAGTTAAAAGTG<br>TGAGGTATCGCCAGGAATTGT	CAGCACGTGGAGCTGTACCAGAAATACAGC	NM_000660.5
**ACTB**	TGCCGACAGGATGCAGAAG<br>GCCGATCCACACGGAGTACT	AGATCAAGATCATTGCTCCTCCTGAGCGC	NM_001101.3

* IFNG was supplied by (Applied Biosystems, Hs00174143_m1)
